# Methodological and ethical challenges in the use of focused ultrasound for blood–brain barrier disruption in neuro-oncology

**DOI:** 10.1007/s00701-023-05782-5

**Published:** 2023-09-06

**Authors:** Santhosh G. Thavarajasingam, John L. Kilgallon, Daniele S. C. Ramsay, Leila Motedayen Aval, Ishaan Ashwini Tewarie, Andreas Kramer, Dannis Van Vuurden, Marike L. D. Broekman

**Affiliations:** 1 Computational Neurosciences Outcomes Center, Department of Neurosurgery, Brigham and Women’s Hospital, Harvard Medical School, Boston, MA USA; 2 Faculty of Medicine, Imperial College London, London, UK; 3 Imperial Brain and Spine Initiative, Imperial College London, London, UK; 4 Department of Neurosurgery, University Medical Centre Mainz, Mainz, Germany; 5 Department of Neurosurgery, Haaglanden Medical Center, The Hague, Netherlands; 6 Princess Maxima Center for Pediatric Oncology, Utrecht, Netherlands; 7 Department of Neurosurgery, Leiden Medical Center, Leiden, Netherlands

**Keywords:** Focused ultrasound, Neuro-oncology, Brain tumor, Glioblastoma, Glioma, IDEAL, SYRCLE, ROBINS-I

## Abstract

**Background:**

Focused ultrasound (FUS) shows promise for enhancing drug delivery to the brain by temporarily opening the blood–brain barrier (BBB), and it is increasingly used in the clinical setting to treat brain tumours. It remains however unclear whether FUS is being introduced in an ethically and methodologically sound manner. The IDEAL-D framework for the introduction of surgical innovations and the SYRCLE and ROBINS-I tools for assessing the risk of bias in animal studies and non-randomized trials, respectively, provide a comprehensive evaluation for this.

**Objectives and methods:**

A comprehensive literature review on FUS in neuro-oncology was conducted. Subsequently, the included studies were evaluated using the IDEAL-D framework, SYRCLE, and ROBINS-I tools**.**

**Results:**

In total, 19 published studies and 12 registered trials were identified. FUS demonstrated successful BBB disruption, increased drug delivery, and improved survival rates. However, the SYRCLE analysis revealed a high risk of bias in animal studies, while the ROBINS-I analysis found that most human studies had a high risk of bias due to a lack of blinding and heterogeneous samples. Of the 15 pre-clinical stage 0 studies, only six had formal ethical approval, and only five followed animal care policies. Both stage 1 studies and stage 1/2a studies failed to provide information on patient data confidentiality. Overall, no animal or human study reached the IDEAL-D stage endpoint.

**Conclusion:**

FUS holds promise for enhancing drug delivery to the brain, but its development and implementation must adhere to rigorous safety standards using the established ethical and methodological frameworks. The complementary use of IDEAL-D, SYRCLE, and ROBINS-I tools indicates a high risk of bias and ethical limitations in both animal and human studies, highlighting the need for further improvements in study design for a safe implementation of FUS in neuro-oncology.

**Supplementary Information:**

The online version contains supplementary material available at 10.1007/s00701-023-05782-5.

## Introduction

Focused ultrasound (FUS) is a non-invasive technology that utilizes high or low-intensity ultrasound waves to treat (mainly) neurological disorders such as Parkinson’s disease and essential tremors but has recently found promising use in neuro-oncology [19, 26]. The first significant *in vivo* application of FUS in animals was conducted by Lynn et al. at Columbia University in 1943 [22]. The use of low-intensity ultrasound (LIFUS) with active scalp cooling [8], and pre-procedural imaging to plan the ultrasonic trajectory has greatly improved the efficacy of FUS since then. Ultimately the relentless scientific efforts led to the first major application of FUS in Neurosurgery dates in 1956 by Ballantine et al. at Massachusetts General Hospital [8, 13].

In the field of glioblastoma (GBM) research, among the most aggressive brain tumors, developments of new neuro-oncological treatment strategies have progressed slowly, since Stupp et al. [39] showed that neurosurgical patients receiving chemoradiation with temozolomide have a median survival of mere 14.6 months. To improve the efficacy of novel drugs, researchers have developed a technique that combines low-intensity focused ultrasound (LIFUS) with intravenously injected gas-encapsulated microbubbles (MBs) to extend the duration of the BBB opening [5]. In addition to enhancing drug delivery, FUS has also been found by pre-clinical studies to enhance neuro-oncological immunotherapy and act as a radiosensitiser [18]. Although most clinical studies have been *in vitro*, FUS is already being used in neurosurgical neuro-oncology, and clinical trials assessing its clinical effect are ongoing [31]. Given that FUS is a relatively novel neurosurgical technology, the question arises whether FUS has been sufficiently scrutinized and undergone evaluation before being used in clinical trials involving humans.

Given the high stakes and narrow margins for error in neurosurgery, particularly for high-risk patients, the evaluation and implementation of surgical devices necessitate strict and meticulous examination. The IDEAL-D framework (Idea, Development, Exploration, Assessment, Long-term study—Devices) has been developed and widely advocated for this purpose. Created by an expert consensus group, the IDEAL-D framework provides a structured approach to the development, evaluation, and implementation of surgical innovations to improve patient outcomes and safety, from first use through clinical practice [10]. However, the adoption of IDEAL(-D) in neurosurgery remains limited [33], likely due to a combination of lack of awareness, insufficient due diligence, and perceptions of the framework as impractical. Nevertheless, to ensure the safety of neurosurgical patients, knowledge of and adherence to the framework are crucial.

Building upon the underpinnings of the IDEAL-D framework and its significance in neurosurgery, the combined application of the SYstematic Review Centre for Laboratory animal Experimentation (SYRCLE) tool and Risk Of Bias In Non-randomized Studies—of Interventions (ROBINS-I) tool emerges as an effective strategy for bolstering the methodological evaluation and uncovering potential biases in various stages of surgical innovation. Using SYRCLE and ROBINS-I together with the IDEAL-D framework allows for a more robust evaluation of the methodological quality and potential biases in studies at various stages of surgical innovation development. SYRCLE is a tool designed to assess the risk of bias in animal studies, while ROBINS-I aims to identify potential biases in non-randomized studies of interventions. Therefore, this article aims to assess whether the IDEAL-D framework has been followed for the introduction of FUS in neuro-oncology, as well as evaluate the methodological quality and risk of bias in studies using SYRCLE and ROBINS-I tools.

## Methodology

A comprehensive narrative literature search was performed searching for all published clinical and preclinical studies, as well as registered trials, on the use of focused ultrasound in neuro-oncology. The search terms used can be found in Supplementary Digital Content Table 1, and the PRISMA flowchart can be found in Supplementary Digital Content Table 2. The search was done using the following databases: “Embase,” “Medline,” “PubMed,” and “ClinicalTrials.gov.” No date limiters were applied. The final search date was December 31, 2022. Inclusion criteria were original studies examining the use of FUS in the context of BBB disruption and enhanced drug delivery in the treatment of brain tumors, clinically or pre-clinically, written in the English language. Reviews were excluded. Data on the various parameters were extracted as per IDEAL framework recommendations on their website idealcollaboration.com [36], a detailed list of the extracted parameters can be found in Supplementary Digital Content Table 3.

To complement the IDEAL-D framework, the SYRCLE and ROBINS-I tools were also employed in our methodology. The SYRCLE tool is specifically designed to assess the risk of bias in animal studies, while the ROBINS-I tool is used for evaluating the risk of bias in non-randomized trials.

Data extraction was conducted using Microsoft Excel, and graphs were created using the R software (version 4.0.4) [35]. In addition, articles were critically appraised, and the risk of bias was determined against all the domains of the SYRCLE and ROBINS-I tool by two independent reviewers (SGT and JK), and a consensus was reached by discussion.

## Results

The literature search yielded 19 original published studies (15 pre-clinical stage 0 studies, two clinical stage 1 studies, and two stage 1/2a) [1, 3, 4, 7, 12, 14, 17, 20, 21, 23, 25, 27–30, 34, 40, 42, 43], and 12 registered trials [16, 37].

### Study and therapy characteristics

**Fig. 1:**
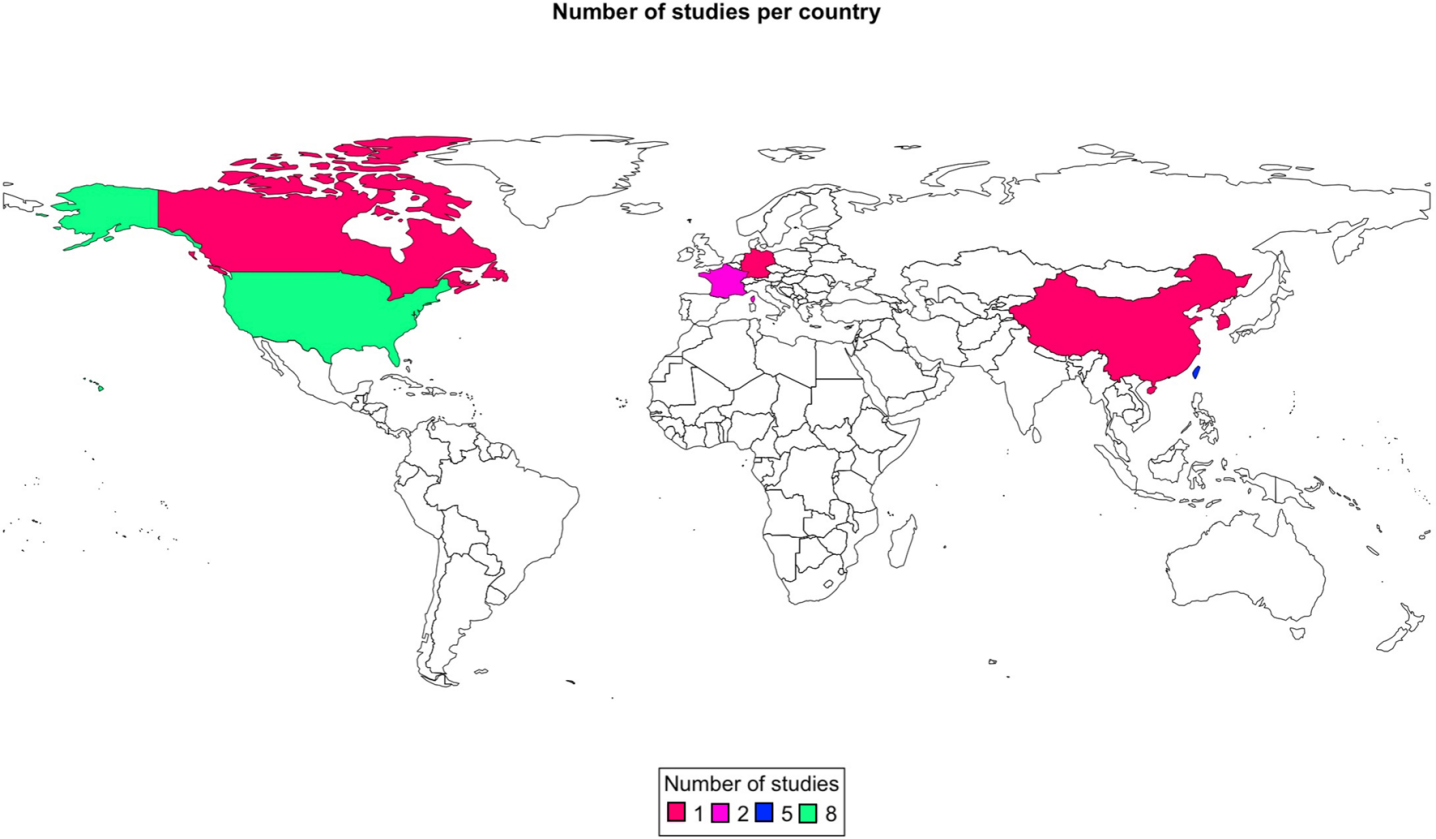
A world map showing the country of origin of the published original studies (*n* = 19) [1, 3, 4, 7, 12, 14, 17, 20, 21, 23, 25, 27–30, 34, 40, 42, 43] on therapeutic FUS for BBB modulation. The legend at the bottom denotes the number of studies published per respective country as separate colour. The corresponding author’s country of affiliation was chosen to represent the origin of the study itself

**Fig. 2:**
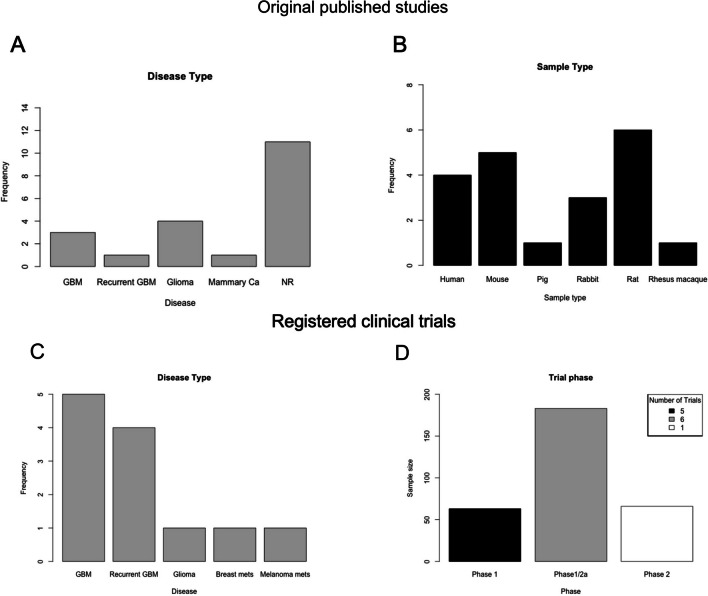
Two bar plots summarise the findings on disease type (**A**) and sample type (**B**) examined by the included published original studies on therapeutic FUS for BBB modulation (*n* = 19) [1, 3, 4, 7, 12, 14, 17, 20, 21, 23, 25, 27–30, 34, 40, 42, 43]. In the second row, two bar plots summarise the findings on disease type (**C**) and trial stage and sample size (**D**) examined by the included registered, but not published, clinical trials on therapeutic FUS for BBB modulation (*n* = 12) [37].

The geographical distribution of the studies is illustrated in Fig. 1, with most studies having been conducted in the USA (*n* = 8). The study characteristics of the original publications are summarised in Table 1 and Fig. 2. The most frequently studied disease types were glioma (not otherwise specified), GBM, and recurrent GBM (Fig. 2A). Rodents were the most commonly used model (Fig. 2B). The most frequently used drugs were Cisplatin (*n* = 3) and Methotrexate (*n* = 4). The ExAblate low-frequency ultrasound system (*n* = 4) and Optison (*n* = 3) were the most used FUS platforms. Registered trials are all in-human, stage 1, or stage 1/2a studies (Table 2). Seven of these trials are marked as “active and recruiting” (Table 2).

**Table 1:** Summary of study characteristics and methodology of published original studies on therapeutic FUS for BBB modulation

Authors	Stage	Country	Disease	Sample size (*n*)	Sample type	Therapy	F/U
Mehier-Humbert et al. (2005)[29]	0	Switzerland	Mammary carcinoma	NR	Rat MAT B III cells (*in vitro*)	**Device**: Sonoporation via 1.15 MHz air backed transducer and a 2.25 MHz FUS transducer combined with US contrast agent (UCA)	None
Kinoshita et al (2006)[17]	0	USA	NR	NR	Mouse brain (*in & ex vivo*)	**Drug**: Herceptin **Device**: 0.69-MHz FUS transducer and the injection of 50 μl of Optison (GE Healthcare) + MRI	None
McDannold et al (2006)[25]	0	USA	NR	9	Rabbit brain (*in & ex vivo*)	**Device**: FUS exposures in conjunction with an US contrast agent (Optison) + passive cavitation detector	None
Treat et al. (2007)[40]	0	USA	NR	2	Rat brain (*in & ex vivo*)	**Drug**: Doxorubicin **Device**: FUS exposure in conjunction with an US contrast agent (Optison)	None
Mei et al. (2009)[30]	0	China	NR	20	Rabbit brain (*in & ex vivo*)	**Drug:** Methotrexate (MTX) **Device**: FUS using in-house–manufactured single-element focused transducer with contrast agent (SonoVue; Bracco Imaging, Geneva, Switzerland) + 1.5-T MRI (Magnetom Symphony; Siemens AG)	None
Liu et al. (2010)[20]	0	USA	NR	NR	Porcine brain and rat brain + human tumor (*in vitro*), cynomol- gus monkey brain (*in & ex vivo*)	**Device**: 3 custom-built, non-focused, piezoelectric transducers (CytoDome Inc., Atlanta, Georgia, USA) with operating frequencies of 85 kHz, 174 kHz, and 1 MHz	None
Liu et al. (2010)[21]	0	Taiwan	NR	NR	Rat brain *(in & ex vivo)* + Rat glioma C6 cells (*in vitro*)	**Device:** MR compatible spherical US transducer (diameter, 60 mm; radius of curvature, 80 mm; frequency, 400 kHz, electric-to-acoustic efficiency, 70%; Imasonics)	None
McDannold et al. (2012)[27]	0	USA	NR	7	Rhesus macaques brain *(in & ex vivo*)	**Device**: ExAblate 4000 low-frequency TcMRgFUS system (InSightec; 30-cm-diameter hemispherical 1,024-element phased array transducer operating at 220 kHz coupled with a 1,024-channel driving system, a treatment planning workstation, and a water cooling/circulation/ degassing system)	None
Ziadloo et al. (2013)[43]	0	USA	NR	NR	Murine SCC (SCCVII) cell line implanted onto female mice (*in & ex vivo*)	**Drug**: naked TNF-alpha plasmid **Device**: custom built, image guided, FUS system, modified from a SonoblateVR 500 (Focus Surgery, Indianapolis, IN; 1 MHz, at a spatial average, temporal peak intensity of 2660W cm–2, using 50 ms pulses, given at a pulse repetition frequency of 1 Hz)	None
Hsu et al. (2013)[12]	0	Taiwan	NR	74	Mouse brain (*in & ex vivo*)	**Drug**: Recombinant adeno-associated viral (rAAV) vectors **Device**: 0.3 mL/kg microbubbles bolus injection (SonoVueH SF6-coated ultrasound microbubbles, mean diameter 2–5 mm, 2.5 mg/kg, Bracco Diagnostics Inc., Milan, Italy; 0.03 mL/kg for clinical diagnostic application); ultrasonic energy was delivered to the brain transcranially using a spherically focused transducer (Imasonics, Besancon, France; diameter = 60 mm, radius of curvature = 80 mm, frequency = 1.5 MHz)	1–6 weeks
Wei et al. (2013)[42]	0	Taiwan	GBM	85	9L rat glioma cell implanted onto mouse brain (*in & ex vivo*)	**Drug**: Temozolomide (TMZ) **Device**: FUS transducer (Imasonics, Besancon, France; diameter = 60 mm, radius of curvature = 80 mm, frequency = 500 kHz) was used to generate concentrated ultrasound energy"—"arbitraryfunction generator (33120A, Agilent, Palo Alto, CA; and DS345, Stanford Research Systems, Sunnyvale, CA) was used to produce the driving signal, which was fed to a radio frequency power amplifier (150A100B, Amplifier Research, Souderton, PA) operating in burst mode"	7–40 days
Alonso et al. (2013)[1]	0	Germany	NR	14	Rat brain (*in & ex vivo*)	**Drug**: Chimeric adeno-associated virus 2/1 (AAV2/1) **Device**: 500 kHz transducer (H-104MR) driven by a function generator (Agilent 33,120 A Function/Arbitrary Waveform Generator; Agilent Technologies, Santa Clara, CA) and amplifier (model 40AD1; AR Amplifier Research, Souderton, PA)	None
Fan et al. (2015)[7]	0	Taiwan	Glioma	NR	Rat brain (*in & ex vivo*)	**Drug**: 1,3-bis(2-chloroethyl)-1-nitrosourea, BCNU **Device**: The first FUS transducer operated at 1 MHz (diameter = 25.4 mm focus length = 52.7 mm; V302, Panametrics, MA, USA) and the second at 10 MHz (diameter = 23.0 mm, focus length = 31.0 mm; SU-110, Sonic Concepts Inc., WA, USA). A function generator (WW2571, Tabor Electronics, Haifa, Israel) created sonication pulses which were amplified with a radio frequency (RF) power amplifier (150A100B, AR, PA, USA) to drive the transducer	6–35 days
Chen et al. (2015)[4]	0	Taiwan	Glioma	50	Healthy and tumor-bearing rats (*in & ex vivo*)	**Drug**: Interleukin-12 **Device**: Focused US transducer (Sonic Concepts, Seattle, WA, USA) + MRI + microbubbles	27–45 days
McDannold et al. (2019)[28]	0	USA	Glioma	23	Healthy and tumor-bearing rats (*in & ex vivo*)	**Drug**: Carboplatin **Device**: ExAblate Neuro low-frequency TcMRgFUS system (InSightec) + MRI + microbubbles	23–53 days
Carpentier A et al. (2016)[3]	1/2a	France	Recurrent GBM	15	Humans	**Drug**: Carboplatin **Device**: 11.5-mm SonoCloud (CarThera) US device + MRI	2–6 months
Mainprize, T et al. (2019)[23]	1	Canada	Glioma	5	Humans	**Drug**: *n* = 1 liposomal doxorubicin, *n* = 4 temozolomide **Device**: ExAblate Neuro (InSightec Tirat Carmel, Israel) system MRgFUS (MRI)	3 months
Idbaih A et al. (2019)[14]	1	France	GBM	19	Humans	**Drug**: Carboplan **Device**: 1-MHz, 11.5-mm diameter cranial US device (SonoCloud-1, CarThera) + MRI	12 months
Park SH et al. (2020)[34]	1/2a	South Korea	GBM	6	Humans	**Drug**: TMZ **Device**: ExAblate low-frequency MRgFUS system (ExAblate Neuro Model 4000 Type 2.0 220 kHz system, InSightec, Haifa, Israel) + MRI + IV microbubble	15.17 ± 1.72 months

**Table 2:** Summary of unpublished, registered clinical trials on therapeutic focussed ultrasound for BBB modulation

NCT	Year	Status	Stage	Disease	Therapy	Primary outcome measure	Sample size (*n*)	Age limit
NCT04804709	2021	Active, not recruiting	1	Diffuse Intrinsic Pontine Glioma Diffuse Pontine and Thalamic Gliomas Diffuse Midline Glioma, H3 K27M-Mutant	**Drug**: Panobinostat **Device**: Focused Ultrasound with neuro-navigator-controlled sonication	AE	3	4—21
NCT03744026	2018	Active, not recruiting	1/2a	Recurrent glioblastoma	**Drug**: Carboplatin (AUC 4–6) **Device**: SonoCloud-9	1) Dose limiting toxicity (DLT) of number of activated ultrasound beams 2) BBB opening will be evaluated by contrast-enhanced T1w magnetic resonance imaging (MR	33	≥ 18
NCT04614493	2020	Active, recruiting	2	Newly diagnosed glioblastoma	**Drug**: Temozolomide **Device**: SonoCloud-9 (SC9) device	PFS	66	≥ 18
NCT04528680	2020	Active, recruiting	1/2a	Recurrent glioblastoma	**Drug**: Albumin-bound paclitaxel (stage 1 and 2), Carboplatin (stage 2) **Device**: Sonication for opening of blood–brain barrier	Dose limiting toxicity (Phase1) 1-year survival rate (Stage 2)	39	≥ 18
NCT04021420	2019	Active, recruiting	1/2a	Metastatic melanoma	**Drug**: Nivolumab **Device**: SONOCLOUD	MSD	21	> 18
NCT03551249	2019	Active, not recruiting	1	Glioblastoma	**Device**: FUS ExAblate, Type 2	AE	20	18—80
NCT03616860	2018	Active, not recruiting	1	Glioblastoma	**Device**: FUS BBB Disruption Exablate Neuro	AE	20	18—80
NCT03714243	2019	Active, recruiting	1	Breast cancer metastases	**Device**: ExAblate Model 4000 Type-2	AE	10	18—80
NCT03712293	2018	Unknown	1	Glioblastoma	**Drug**: Temozolomide **Device**: ExAblate Type 2.0 BBBD	AE	10	19—80
NCT04440358	2020	Active, recruiting	1/2a	Recurrent glioblastoma	**Drug**: Carboplatin **Device**: ExAblate Type 2.0 BBBD	AE	50	18 -80
NCT04417088	2020	Active, recruiting	1/2a	Recurrent glioblastoma	**Drug**: Carboplatin **Device**:ExAblate Type 2.0 BBBD	AE	30	18—80
NCT04446416	2020	Active, recruiting	1/2a	Recurrent glioblastoma	**Drug**: Bevacizumab **Device**: NaviFUS System	AE & PFS at 6 months	10	18—80

### Outcomes and complications

The primary outcomes of all included studies were focused on investigating the efficacy and safety of BBB disruption using FUS. However, we observed a high degree of heterogeneity among the specific outcome measures used across the studies (Table 3). Of the included studies, five investigated the survival and safety of FUS in BBB disruption, while 11 studies examined the effect of BBB on macromolecular transfer, including medication transfer across the BBB (Table 3). Most animal studies (*n* = 10) do not report functional outcomes, as they were generally sacrificed after the procedure for histological examination. Interestingly, most published clinical and pre-clinical studies (*n* = 12) did not report complications, while only five studies reported no complications (Table 3). Nonetheless, most included animal and human studies reported largely positive outcomes in terms of their respective primary and secondary outcomes (*n* = 17). None of the in-human studies reported direct side effects or complications of FUS therapy. Overall, BBB disruption was reported to be associated with successful disruption of the BBB, increased drug delivery, improved survival rates, and good safety (Table 3).

**Table 3:** Summary of main findings of all published original studies on therapeutic FUS for BBB modulation

Authors	Primary & secondary outcomes	Main findings	Complications and SE
Mehier-Humbert et al. (2005)[29]	1) Size of the pores; 2) Duration of pore opening	1) Internalization of molecules with diameters up to 37 nm using sonification was efficient and occurred with little complications, but 75 nm particles entered only a few cells	NR
Kinoshita et al (2006)[17]	1) Leakage through the BBB; 2) Herceptin delivered through the BBB; 3) Histological damage	1) Herceptin can be delivered into the mouse CNS through the BBB employing an MRI-guided FUS; 2) Amount of Herceptin delivered correlated with extent of barrier opening; 3) Few adverse histological changes	1) None
McDannold et al (2006)[25]	1) Association of cavitation activity with the induction of blood–brain barrier disruption (BBBD)	1) BBBD resulting from focused ultrasound pulses in the presence of Optison occur without indicators for inertial cavitation in vivo, wideband emission and extravasation	NR
Treat et al. (2007)[40]	1) Successful BBB disruption; 2) Fluorometric measurements of DOX; 3) Histological damage; 4) Correlation of MRI signal and DOX delivery	1) Targeted delivery by focused ultrasound may make DOX chemotherapy a viable treatment option against CNS tumors; 2) MRI signal enhancement in the sonicated region correlated strongly with tissue DOX concentration	1) Severe macroscopic tissue damage in rats administered 0.5 mL/kg Optison
Mei et al. (2009)[30]	1) Optimal exposure time for reversible BBB disruption; 2) Comparison to cohort with internal carotid artery (ICA) injections of MTX	1) MTX concentration in the sonicated group notably higher than that in the IV control group and ICA group (p < 0.01)	1) Degree of vascular effects and tissue necrosis correlated with increased exposure
Liu et al. (2010)[20]	1) Successful transport of large molecules across brain tissue in vitro; 2) Validation by examining the effect of US in monkey brain in vivo	1) Exposure to ultrasound at various frequencies (85 kHz, 174 kHz, and 1 MHz) enhanced the permeation of tritiumlabeled molecules with molecular weight up to 70 kDa across porcine brain tissue	NR
Liu et al. (2010)[21]	1) Successful delivery of macromolecular therapeutic agents to the CNS; 2) Ideal use of technology	1) FUS can temporarily disrupt the BBB, increasing local EPR in the CNS; 2) This technology is ideally suited for transcranial delivery of drugs with molecular weights greater than 400 Da	1) Penetration is hampered at molecular weights of 2,000 kDa
McDannold et al. (2012)[27]	1) Identification of a safety window for BBB disruption without evident tissue damage	1) Study shows feasibility of reliably and repeatedly inducing focal BBB disruption without significant vascular or brain tissue damage in a clinically-relevant animal model using a TcMRgFUS system designed for human use	NR
Ziadloo et al. (2013)[43]	1) Effect of exposure on tumor growth	1) Exposures alone had no effect on tumor growth; 2) Significant growth inhibition was observed with injection of TNF-a plasmid, tumor growth was inhibited with pFUS	NR
Hsu et al. (2013)[12]	1) Successful delivery of AAV2 vector and GFP expression	1) IV-administered AAV2-GFP (green fluorescence protein) with a low viral vector titer (16109 vg/g) can successfully penetrate the BBB-opened brain regions to express GFP	NR
Wei et al. (2013)[42]	1) Effects of treatment on tumor progression, animal survival, and brain tissue histology	1) Compared to TMZ alone, combined FUS treatment increased the TMZ CSF/plasma ratio from 22.7% to 38.6%, reduced the 7-day tumor progression ratio from 24.03 to 5.06, and extended the median survival from 20 to 23 days	NR
Alonso et al. (2013)[1]	1) Successful opening of the BBB, gene transfer, and expression rate in transduced cells	1) Chimeric adeno-associated virus 2/1 (AAV2/1) particles containing the coding region for the LacZ gene were efficiently delivered into the rat brain upon IV administration after BBB FUS opening with vascular acoustic resonators	NR
Fan et al. (2015)[7]	1) Successful FUS-induced drug release by acoustic emission; 2) In vivo BBB opening by FUS exposure with BCNU bubbles	1) Excitation of BCNU bubbles by FUS resulted in stable cavitation, significantly reduced the occurrence of hazards of exposure; 2) BCNU bubbles with FUS showed control of tumor progression, improved survival from 26 to 35 days	NR
Chen et al. (2015)[4]	1) Efficacy in inducing BBB opening; 2) Effect on tumor progression; 3) Effect of the immune response	1) BBB opening had no effect on T lymphocytes; 2) IL-12 administration triggered increase in all TIL populations; 3) Combined FUS-BBB opening with IL-12 administration produced most significant IL-12 increase	NR
McDannold et al. (2019)[28]	1) Safety; 2) Efficiency of opening BBB; 3) Survival	1) Tumor volume doubling time FUS and carboplatin in rats increased by 96% and 126% compared to rats that received carboplatin alone and non-sonicated controls; 2) Increases in median survival were 48% and 66%	1) None
Carpentier A et al. (2016)[3]	1) Feasibility of repeated transient opening of the BBB by pulsed US; 2) Safety of procedure with recurrent GBM before receiving chemotherapy	1) Contrast-enhanced MRI indicated that the BBB was disrupted at acoustic pressure levels up to 1.1 megapascals without detectable adverse effects on radiologic or clinical examination	1) None
Mainprize, T et al. (2019)[23]	1) Safety and feasibility of opening the BBB in brain tumor patients using MRgFUS; 2) Feasibility of chemotherapy delivery	1) The BBB within the target volume showed radiographic evidence of opening with an immediate 15–50% increased contrast enhancement; 2) Biochemical analysis suggest chemotherapy delivery is safe and feasible	1) None
Idbaih A et al. (2019)[14]	1) Safety and tolerance to sonication with the SonoCloud-1 device; 2) Disruption of the BBB using the SonoCloud-1 system; 3) PFS and the OS of the patients treated with SonoCloud-1 device; 4) Biocompatibility of the device; 5) Practical feasibility for future trials	1) Patients with no or poor BBB disruption visible on MRI had a median progression-free survival (PFS) of 2.73 months, and a median overall survival (OS) of 8.64 months; 2) Patients with clear BBB disruption had a median PFS of 4.11 months, and a median OS of 12.94 months	NR
Park SH et al. (2020)[34]	1) Survival; 2) Recurrence; 3) MRgFUS related complications	1) Survival rate up to 1 year was 100%	1) No short- or long-term complications from BBBD; 2) Two patients (of six) had GBM recurrence

## Stage 0 studies

### IDEAL-D analysis

**Fig. 3:**
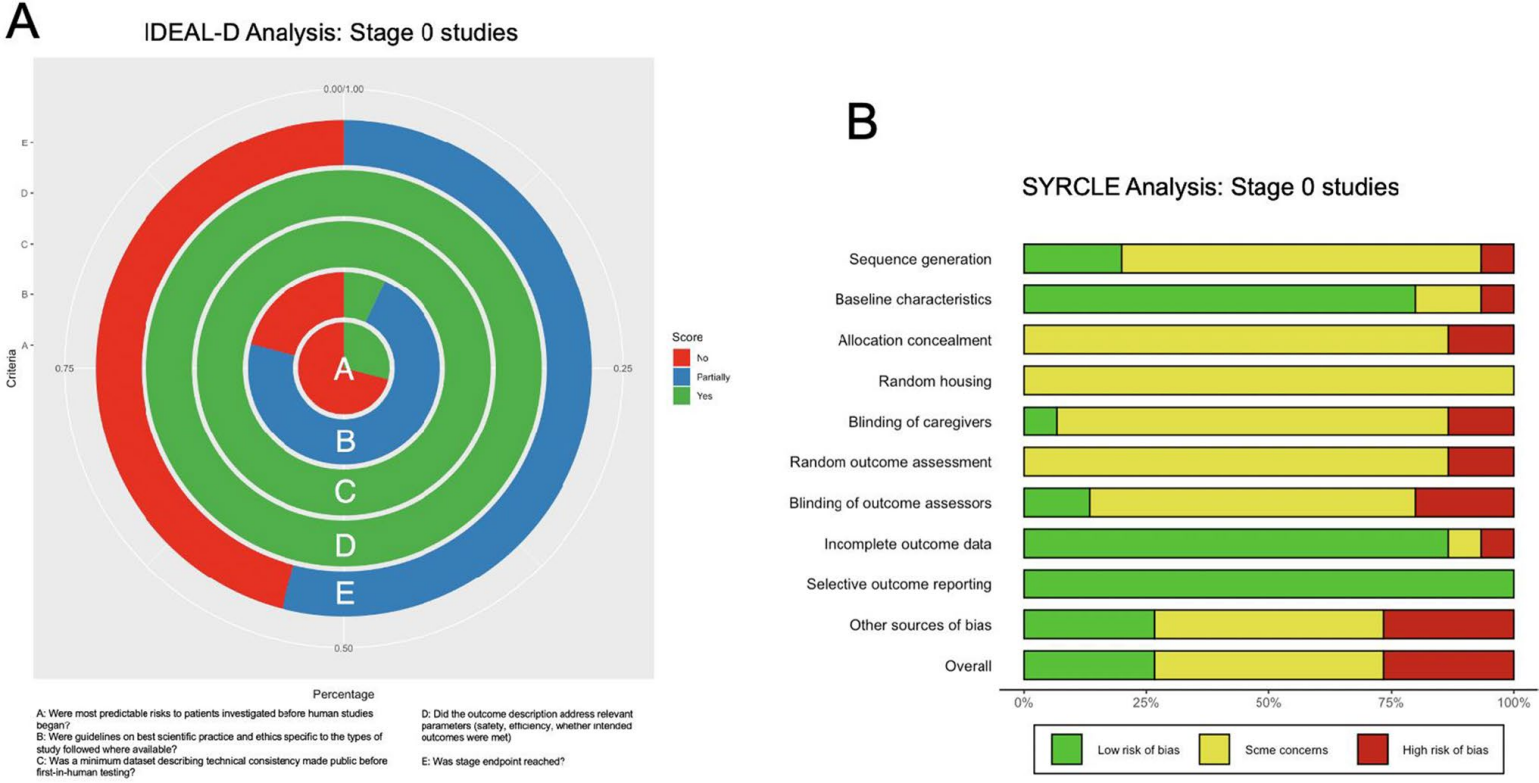
**A** A stacked pie chart summarised the findings of the IDEAL-D analysis of published stage 0 studies (*n* = 15) [1, 4, 7, 12, 17, 20, 21, 25, 27–30, 40, 42, 43]. Each ring is denoted with an alphabetical letter and the corresponding categorical variable is denoted in the legend at the bottom of the graph. A) Were all predictable risks to patients investigated before human studies began? B) Were guidelines on best scientific practice and ethics specific to the types of study followed where available? C) Was a minimum dataset describing technical consistency made public before first-in-human testing? D) Did the outcome description address relevant parameters (i.e. Whether intended goal of procedure is accomplished? Level of difficulty of performing procedure or using device as compared to standard of care? Safety risks? Desirability of intervention?) E) Was stage endpoint reached? (Any studies that could avoid predictable risks of failure or harm to the first human should have been conducted.) The colour of the rings correlate to the legend at the right side of the graph and denote whether the categorical variables were fully addressed (“Yes”), incompletely addressed (“Partially”), or not at all addressed (“No”). **B** Risk of bias summary plot for non-randomized studies with a bar chart of the distribution of risk-of-bias judgments for all included stage 0 studies (*n* = 15) [1, 4, 7, 12, 17, 20, 21, 25, 27–30, 40, 42, 43] across the domains of the SYRCLE tool, shown in percentages (%), is shown. At the bottom, an overall risk of bias, which represents the collated risk-of-bias judgements for all domains, is depicted

IDEAL-D stage 0 studies involve the preclinical stage of surgical innovation development, focusing on laboratory and animal research, and aim to establish the feasibility, safety, and proof-of-concept for the new technique or device before transitioning to clinical testing (Fig. 3) [10].

**Fig. 4:**
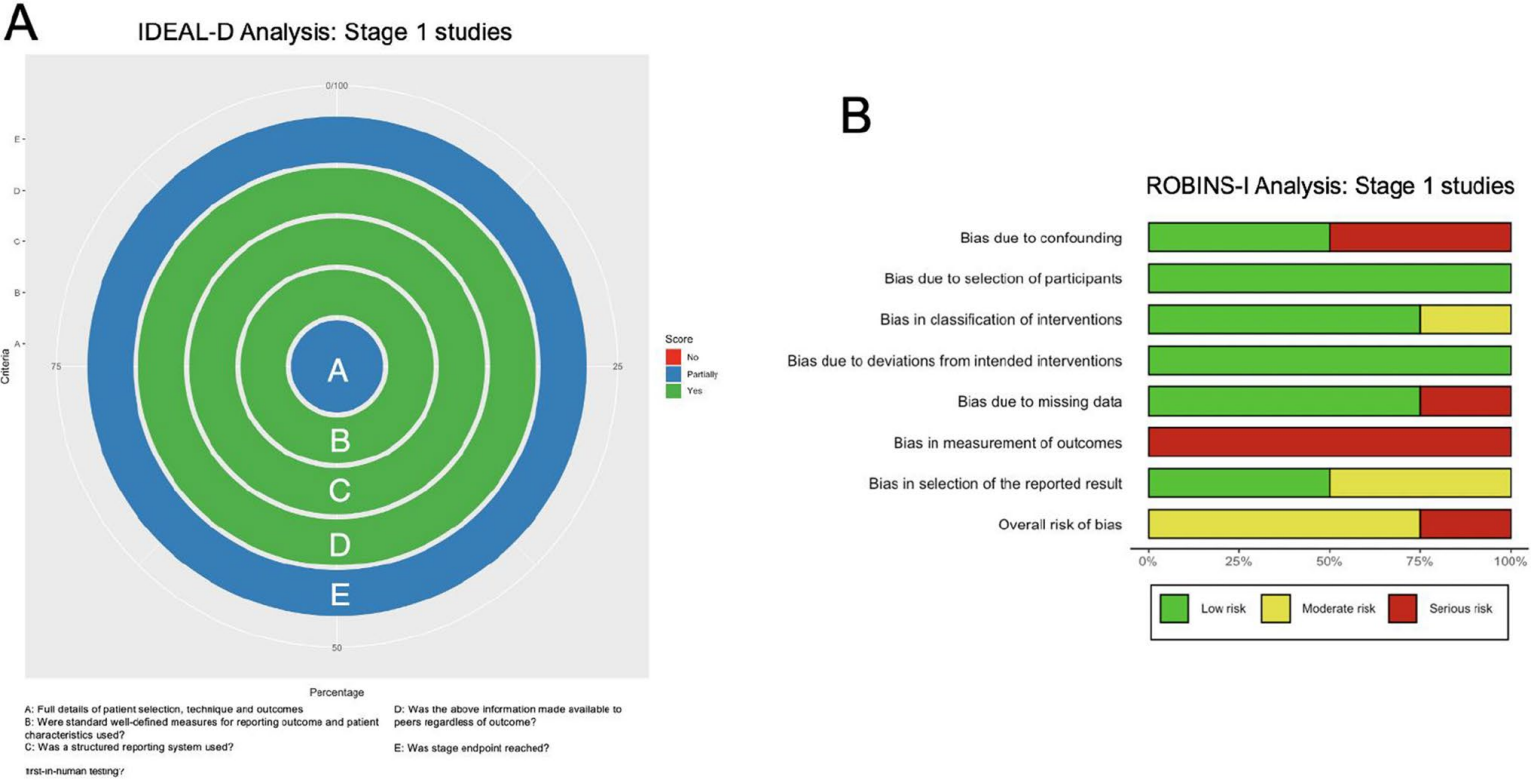
**A** stacked pie chart summarised the findings of the IDEAL-D analysis of published stage 1 (excluding stage 1/2a) studies (*n* = 2) [14, 23]. Each ring is denoted with an alphabetical letter and the corresponding categorical variable is denoted in the legend at the bottom of the graph. A) Were full details of patient selection, technique, and outcomes and patients not selected during the time frame, and why provided? B) Were standard well-defined measures for reporting outcome and patient characteristics used? C) Was a structured reporting system used? D) Was the above information made available to peers regardless of outcome? E) Was stage endpoint reached? (Outcomes will determine whether to proceed to stage 2a.) The colour of the rings correlate to the legend at the right side of the graph and denote whether the categorical variables were fully addressed (“Yes”), incompletely addressed (“Partially”), or not at all addressed (“No”). **B** Risk of bias summary plot for non-randomized studies with a bar chart of the distribution of risk-of-bias judgments for all included stage 1 studies (*n* = 2) [3, 34] across the domains of the ROBINS-I tool, shown in percentages (%) is shown. At the bottom, an overall risk of bias, which represents the collated risk-of-bias judgements for all domains, is depicted

Of the 15 studies [1, 4, 7, 12, 17, 20, 21, 25, 27–30, 40, 42, 43] included in the analysis only 4 studies [1, 21, 27, 43] investigated the predictable risks in animal models, while the remaining studies did not address these risks adequately. Most of the studies were scored negatively regarding the exploration of the majority of predictable risks to patients due to the lack of investigation of complex neurological sequelae in the animals, including testing of neurological higher functioning. Among the included studies, Alonso et al. [42] (2013) stood out for following guidelines on best scientific practises and ethics specific to animal studies [11, 32]. None of the other studies, reported on the use of randomization nor participant and investigator blinding. However, all studies made a minimum dataset describing technical consistency before first-in-human testing and addressed relevant parameters as outlined in the methodology section (*n* = 15/15). Overall, none of the studies fully reached the stage endpoint due to poor methodology, and only six studies partially reached it (Fig. 4A). All scores are also presented in Supplementary Digital Content Table 4.

Fifteen animal studies were evaluated for their adherence to animal welfare and ethics guidelines. Of the 15 studies, six had formal approval from an animal committee [4, 17, 25, 28, 42, 43], five did not have formal approval but followed appropriate animal care policies [7, 12, 20, 30, 40], and four had no information regarding approval or animal care [1, 21, 27, 29]. Regarding euthanization and anesthesia methods, all studies except one [42] reported appropriate methods. There was a lack of information provided on the level of animal care in most studies. All studies were transparent regarding any conflicts of interest including received grants.

### SYRCLE analysis

**Fig. 5:**
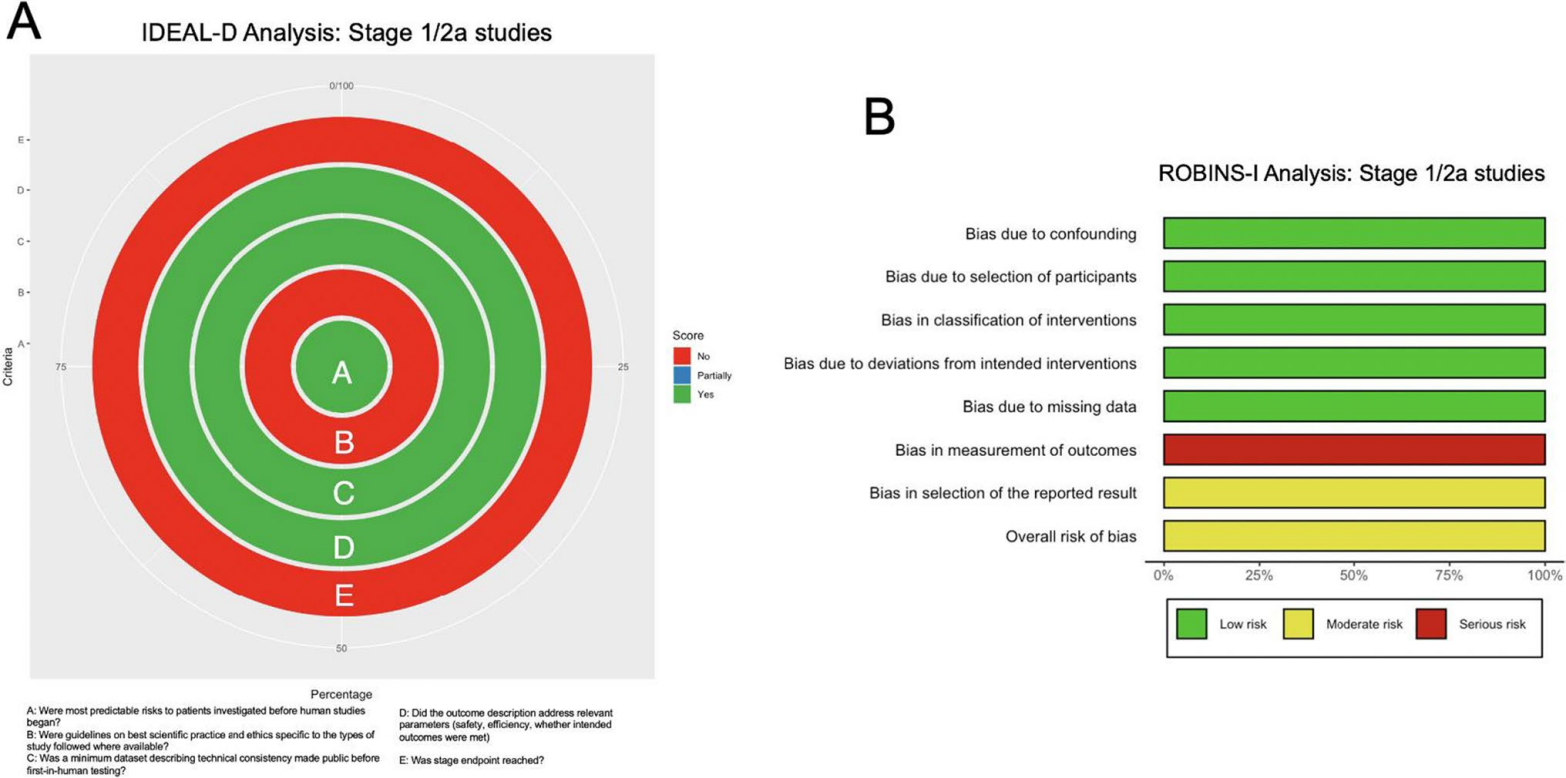
**A** A stacked pie chart summarised the findings of the IDEAL-D analysis of published stage 1/2a studies (*n* = 2) [3, 34]. Each ring is denoted with an alphabetical letter and the corresponding categorical variable is denoted in the legend at the bottom of the graph. A) Was a study protocol made available? B) Were standard well-defined measures for reporting outcome and patient characteristics used? C) Were all exclusions reported and explained? D) Were all cases reported sequentially with annotation and explanation of when and why changes to indication or procedure took place? E) Was stage endpoint reached? (Display main outcomes graphically to illustrate the above.) The colour of the rings correlate to the legend at the right side of the graph and denote whether the categorical variables were fully addressed (“Yes”), incompletely addressed (“Partially”), or not at all addressed (“No”). **B** Risk of bias summary plot for non-randomized studies with a bar chart of the distribution of risk-of-bias judgments for all included stage 1/2a studies (*n* = 2) [3, 34] across the domains of the ROBINS-I tool, shown in percentages (%) is shown. At the bottom, an overall risk of bias, which represents the collated risk-of-bias judgements for all domains, is depicted

SYRCLE analysis of 15 studies, presented in Fig. 5 and Supplementary Digital Content Table 5, showed varying degrees of bias across different parameters. Only three studies [7, 28, 43] exhibited a low risk of bias in sequence generation by properly randomizing samples, while the remaining 12 [1, 4, 12, 17, 20, 21, 25, 27, 29, 30, 40, 42] showed concerns or unclear risk, mainly due to lack of randomization. One study [29] notably scored high risk for not employing controls. For baseline characteristics, most studies (12/15) scored low risk of bias; two showed some concerns due to insufficient animal data. Allocation concealment was a concern in most studies (*n* = 13/15), with two scoring high risk. All studies scored “some concerns” for random housing, with the majority (12/15) not addressing the blinding of caregivers. Only Alonso et al. [42] implemented a low-risk, double-blinding methodology. Random outcome assessment was unclear in most (*n* = 13/15) studies, with two scoring high risk. Outcome assessor blinding was generally unaddressed (*n* = 10/15), except for two studies [1, 28]. Incomplete outcome data had low risk in most cases (*n* = 13/15), with one high-risk exception. All studies scored low risk for selective outcome reporting. Overall, the studies varied in bias risk: four high, seven unclear, and four low. An in-depth explanation of scoring parameters and rationale is provided in Supplementary Digital Content Table 5.

## Stage 1 studies

### IDEAL-D analysis

Under the IDEAL-D framework [3, 34], stage 1 studies mark the initial foray into human clinical testing, aiming to assess the safety and feasibility of the innovation within a confined patient cohort.

Two studies were included in the analysis (Fig. 4A) [14, 23], both of which had well-defined measures for reporting outcomes and patient characteristics, a structured reporting system, and disclosed all information regardless of outcomes. They partially reached the stage endpoint due to methodological shortcomings, particularly the lack of blinding and randomization. Ethical standards were maintained with formal board approval and informed patient consent, but patient confidentiality procedures were not explicitly described. Both studies fully disclosed any potential conflicts of interest. All scores are also presented in Supplementary Digital Content Table 6.

### ROBINS-I analysis

ROBINS-I analysis, summarised in Fig. 4B and Supplementary Digital Content Table 7, of the same two studies, showed a low risk of bias for participant selection, deviations from intended interventions, and selection of the reported result. However, they scored high risk due to confounding factors: heterogenous medication regime for Mainprize et al. [23] and a varied patient sample for Idbaih A et al. [14]. Mainprize et al. [23] also had a moderate risk for classification of intervention and high risk for missing data due to a high dropout rate and missing tumor samples. Both studies had a high risk of bias in outcome measurement due to a lack of blinding. Overall, Mainprize et al [23]. was assessed as having a high risk of bias, while Idbaih A et al. [14] was seen as a moderate risk.

## Stage 1/2a studies

### IDEAL-D analysis

IDEAL-D stage 1/2a studies [3, 34] are situated a transitional stage between early clinical safety evaluations and the exploration of the novel surgical technique or device in a larger patient cohort. These studies involve refining the technique, determining optimal parameters, and expanding evaluations to multiple centers for more robust safety and efficacy data. Two studies were included in the analysis (Fig. 5A). All scores are also presented in Supplementary Digital Content Table 8.

The analysis revealed that both studies scored positively for making their study protocol available, explaining all exclusions, and reporting all cases sequentially with annotation and explanation of when and why changes to indication or procedure took place. However, the studies were only partially able to reach the stage endpoint of the IDEAL-D framework due to poor outcome reporting methodology. Both stage 1/2a studies obtained formal approval from their respective ethics boards and respected patient autonomy by providing information about the study and gaining informed consent. The studies both ensured patient safety and disclosed any potential conflicts of interest. However, no information regarding the confidentiality of patient data was available.

### ROBINS-I analysis

In Fig. 5B and Supplementary Digital Content Table 9, the results of the ROBINS-I analysis are shown (*n* = 2). The studies [3, 34] demonstrated a low risk of bias in several categories, such as confounding, participant selection, intervention classification, deviations from intended interventions, and missing data. However, they scored highly for bias in outcome measurement due to insufficient description of how neurological outcomes were measured, and moderate risk of other bias due to a lack of blinding. Overall, both studies were classified as having a moderate risk of bias.

## Discussion

This methodological analysis of the 15 pre-clinical studies revealed that none fully and only six partially reached the IDEAL stage endpoint for stage 0 studies. Both IDEAL-D stage 1 studies only partially reached the stage endpoint due to poor methodology of blinding and randomization. Of the two stage 1/2a studies, poor outcome reporting meant that the stage endpoint was only partially reached. However, all stage 1 and 1/2a studies stated that they obtained formal approval from their respective ethics boards, respected patient autonomy, and ensured patient safety. Overall, the SYRCLE analysis scored four pre-clinical studies as high, seven studies as unclear, and four studies as low risk of bias. The ROBINS-I analysis for clinical studies showed that the stage 1 studies were scored as moderate (*n* = 1) and high risk of bias (*n* = 1), and both stage 1/2a studies were classified as having a moderate risk of bias. Given our methodological findings, the efficacy and safety of BBB disruption using FUS, as summarized in our review, are likely subject to overestimation and lack of generalizability.

The results of the IDEAL-D and SYRCLE analyses suggest that most stage 0 studies investigating FUS in animal models are conducted with poor methodology and a high risk of bias. Specifically, most studies failed to use appropriate animal models, follow guidelines on best scientific practice and ethics specific to animal studies, or adequately address predictable risks to patients, including complex neurological sequelae and testing of neurological higher functioning. The SYRCLE analysis revealed that sequence generation parameters, including baseline characteristics, allocation concealment, random housing, and blinding of caregivers and outcome assessors, were not addressed. The absence of formal animal committee approval in several studies demonstrates a lack of awareness of ethical guidelines by researchers. This is cause for concern as unnecessary animal suffering may jeopardize scientific research efforts and undermine study credibility. Although most studies reported appropriate detail regarding euthanization and methods for anesthesia, information about animal living conditions was frequently omitted. These findings highlight the need for more rigorous methodological standards and adherence to best scientific practice and ethics in stage 0 studies of FUS in animal models.

Our findings suggest that the animal studies have not yet reached the IDEAL-D stage endpoint, indicating that more high-quality non-human studies are necessary to ensure the safety of FUS before translation into humans [24]. However, many human clinical trials have already been registered, and the feasibility of halting them is limited. Thus, increased methodological scrutiny of human studies to ensure the safety and efficacy of FUS is required. Several studies explore the complex neurological side-effects of FUS; however, the lack of bias and randomization in stage 1 and stage 1/2a undermine the reliability and validity of the findings [6]. The current study serves as an important stimulus for employing appropriate measures to ensure the high validity of findings, such as pre-specified endpoints, blinding, and randomization. This would lead to improved reliability of study results and increase the safety of FUS technology for human subjects.

Overall, our results demonstrate a concerning lack of adherence to methodological and ethical frameworks such as IDEAL-D, SYRCLE, and ROBINS-I in studies related to FUS in neurosurgery, with none of the studies fully reaching the IDEAL end stage [11]. One possible explanation for the lack of adherence to methodological and ethical frameworks is a lack of awareness among researchers [8]. While IDEAL-D was developed in the UK, our analysis found that most included studies on FUS were from the USA. This suggests that there may be a need to increase awareness of these frameworks outside of Europe and to encourage researchers to incorporate them into their study protocols [41]. Most studies report positive outcomes of FUS accompanied by few or no complications associated with the technology; however, the lack of adherence to ethical and methodological frameworks, as noted in the analysis, places the reliability and validity of the results obtained into question. Therefore, the efficacy and safety of BBB disruption using FUS, which our study also summarised, may lack accuracy and generalizability and could be a misleading overestimation. Therefore, further research that adheres to ethical and methodological frameworks, such as SYRCLE, ROBINS-I, and IDEAL-D, is needed to confirm the efficacy and safety of FUS for brain tumors and to guide clinical practice.

The present study’s findings may be limited by several factors. While the study used three different appraisal tools, they all have limitations, such as the ROBINS-I tools’ inability to capture all sources of bias [38] and the IDEAL-D tools’ subjective nature [24]. The retrospective application of the IDEAL-D framework in this study may be challenging due to its inherent limitations. The IDEAL-D framework is designed to guide the planning, conduct, and reporting of surgical innovation studies, and applying it retrospectively may present challenges in accurately assessing the study’s methodology and results—it is similar to trying “to put the genie back inside the bottle”. Furthermore, the limited availability of data in some studies may have hindered the ability to assess certain outcomes or biases [9], and the relatively small sample size of studies included may limit the generalizability of the findings [2]. Moreover, publication bias may have affected the study’s results, as studies with positive outcomes may be more likely to be published than those with negative outcomes [15]. Despite these limitations, the present study provides important insights into the use of FUS and highlights areas where further research is needed.

## Conclusion

In conclusion, our narrative analysis of FUS studies in the field of neuro-oncology has uncovered important concerns about the ethical and methodological foundations of this emerging technology. Our detailed evaluation reveals a potential for bias and a concerning degree of methodological inconsistency, issues which have the potential to significantly compromise the validity of reported safety and efficacy outcomes. Particularly concerning is the noted deficit in compliance with recognized methodological and ethical standards, including the IDEAL-D, SYRCLE, and ROBINS-I frameworks. Animal studies were particularly found wanting in this regard, necessitating a renewed commitment to ethical and methodological rigor to ensure robust results that can effectively inform subsequent clinical trials. Given the considerable number of registered human clinical trials, the urgent requirement for improvements in study quality cannot be overstated. Further, the noted geographical disparity in adherence to frameworks like IDEAL-D makes a compelling case for a global initiative to standardize research methodologies. Future research in this field must work assiduously to address these shortcomings, uphold stringent ethical norms, and refine trial methodologies. This is crucial to ensure that the implementation of FUS in neuro-oncology practice is both safe and effective.

### Supplementary Information

Below is the link to the electronic supplementary material.

Supplementary file1 (DOCX 514 KB)

## Data Availability

Provided in the supplementary file.

## Code availability

Available on request.

## Abbreviations

BBB: Blood-brain barrier
FUS: Focused ultrasound
IDEAL: Idea, Development, Exploration, Assessment, Long-term follow-up
IDEAL-D: IDEAL Description, an expanded version of IDEAL
ROBINS-I: Risk Of Bias In Non-randomized Studies—of Interventions
SYRCLE: Systematic Review Centre for Laboratory animal Experimentation
UK: United Kingdom
US: United States
WHO: World Health Organization
