# Supplementary material for: Methodological and ethical challenges in the use of focused ultrasound for blood–brain barrier disruption in neuro-oncology
Source: Acta Neurochir (Wien). 2023 Sep 6;165(12):4259–77. doi: 10.1007/s00701-023-05782-5 (PMC10739192; doi:10.1007/s00701-023-05782-5)
Supplement: Supplementary file 1 — Supplementary file1 (DOCX 514 KB) [file 701_2023_5782_MOESM1_ESM.docx]

***Supplemental Digital Content***

**Methodological and ethical challenges**

**in the use of focused ultrasound**

**for blood-brain barrier disruption**

**in neuro-oncology**

**AUTHORS:**

Santhosh G. Thavarajasingam^1,2,3^ ;

John L. Kilgallon^1^, Daniele S. C. Ramsay^2,3^

Leila Motedayen Aval^2,3^ ; Ishaan Ashwini Tewarie^1,4^ ; Andreas Kramer^5^ ;

Dannis Van Vuurden^6^  ; Marike L. D. Broekman^4,7^

**INSTITUTION:**

1. Computational Neurosciences Outcomes Center, Department of Neurosurgery, Brigham and Women's Hospital, Harvard Medical School, Boston, Massachusetts.
2. Faculty of Medicine, Imperial College London, London, United Kingdom.
3. Imperial Brain and Spine Initiative, Imperial College London, London, United Kingdom.
4. Department of Neurosurgery, Haaglanden Medical Center, The Hague, Netherlands.
5. Department of Neurosurgery, University Medical Centre Mainz, Mainz, Germany.
6. Princess Maxima Center for Pediatric Oncology, Utrecht, Netherlands.
7. Department of Neurosurgery, Leiden Medical Center, Leiden, Netherlands.

**Tables of Content**

[**Supplementary Table 1:** The search strategy employed in this study^1^. 3](#_Toc140780849)

[**Supplementary Table 2:** Prisma flowchart of our review. 6](#_Toc140780850)

[**Supplementary Table 3**: A full list of extracted parameters^1^. 7](#_Toc140780851)

[**Supplementary Table 4**: Scoring on IDEAL-D tool for Phase 0 studies. 9](#_Toc140780852)

[**Supplementary Table 5**: Scoring on SYRCLE tool for Phase 0 studies^17^. 10](#_Toc140780853)

[**Supplementary Table 6**: Scoring on IDEAL-D tool for Phase 1 studies. 13](#_Toc140780854)

[**Supplementary Table 7**: Scoring on ROBINS-1 tool for Phase 1 studies^20^. 13](#_Toc140780855)

[**Supplementary Table 8**: Scoring on IDEAL-D tool for Phase 1 studies. 14](#_Toc140780856)

[**Supplementary Table 9**: Scoring on ROBINS-1 tool for Phase 1 studies. 14](#_Toc140780857)

[**References** 15](#_Toc140780858)

#

# **Supplementary Table 1:** The search strategy employed in this study.

| **Database** | **Search terms** | **Publication dates** | **Results (n)** |
| --- | --- | --- | --- |
| Medline | ((Focused Ultrasound or FUS or LIFUS or LIFU or HIFU or HIFUS or MRgFUS or FUS-LBx or High-Intensity Focused Ultrasound or Low-Intensity Focused Ultrasound or Magnetic Resonance-guided Focused Ultrasound) and (brain tumor or brain tumour or neuro-oncology or glioma or glioblastoma or meningioma or GBM or brain neoplasm or cerebral tumor or cerebral tumour or Craniopharyngioma or Meningioma or Oligodendroglioma or Pituitary tumor or brain cancer or intracranial tumor or intracranial neoplasm or brain carcinoma or astrocytoma or medulloblastoma or ependymoma or acoustic neuroma or pinealoma or choroid plexus tumor or neuroblastoma or brain metastases)).af. | 1946 - December 2022 | n = 358 |
| Embase | ((Focused Ultrasound or FUS or LIFUS or LIFU or HIFU or HIFUS or MRgFUS or FUS-LBx or High-Intensity Focused Ultrasound or Low-Intensity Focused Ultrasound or Magnetic Resonance-guided Focused Ultrasound) and (brain tumor or brain tumour or neuro-oncology or glioma or glioblastoma or meningioma or GBM or brain neoplasm or cerebral tumor or cerebral tumour or Craniopharyngioma or Meningioma or Oligodendroglioma or Pituitary tumor or brain cancer or intracranial tumor or intracranial neoplasm or brain carcinoma or astrocytoma or medulloblastoma or ependymoma or acoustic neuroma or pinealoma or choroid plexus tumor or neuroblastoma or brain metastases)).af. | 1974 - December 2022 | n = 915 |
| Pubmed | ((Focused Ultrasound or FUS or LIFUS or LIFU or HIFU or HIFUS or MRgFUS or FUS-LBx or High-Intensity Focused Ultrasound or Low-Intensity Focused Ultrasound or Magnetic Resonance-guided Focused Ultrasound) and (brain tumor or brain tumour or neuro-oncology or glioma or glioblastoma or meningioma or GBM or brain neoplasm or cerebral tumor or cerebral tumour or Craniopharyngioma or Meningioma or Oligodendroglioma or Pituitary tumor or brain cancer or intracranial tumor or intracranial neoplasm or brain carcinoma or astrocytoma or medulloblastoma or ependymoma or acoustic neuroma or pinealoma or choroid plexus tumor or neuroblastoma or brain metastases)).af. | 1991 – December 2022 | n = 1522 |
| ClinicalTrials.gov | Focused ultrasound \(FUS\) | 1943 – August 2022 | n = 33 |

In Supplementary Table 1 the search strategy performed on 31 December 2022 is shown below outlining the respective databases, the search terms, publication dates chosen as limiting factors, and number of results from each database are shown.

# **Supplementary Table 2:** Prisma flowchart of our review.


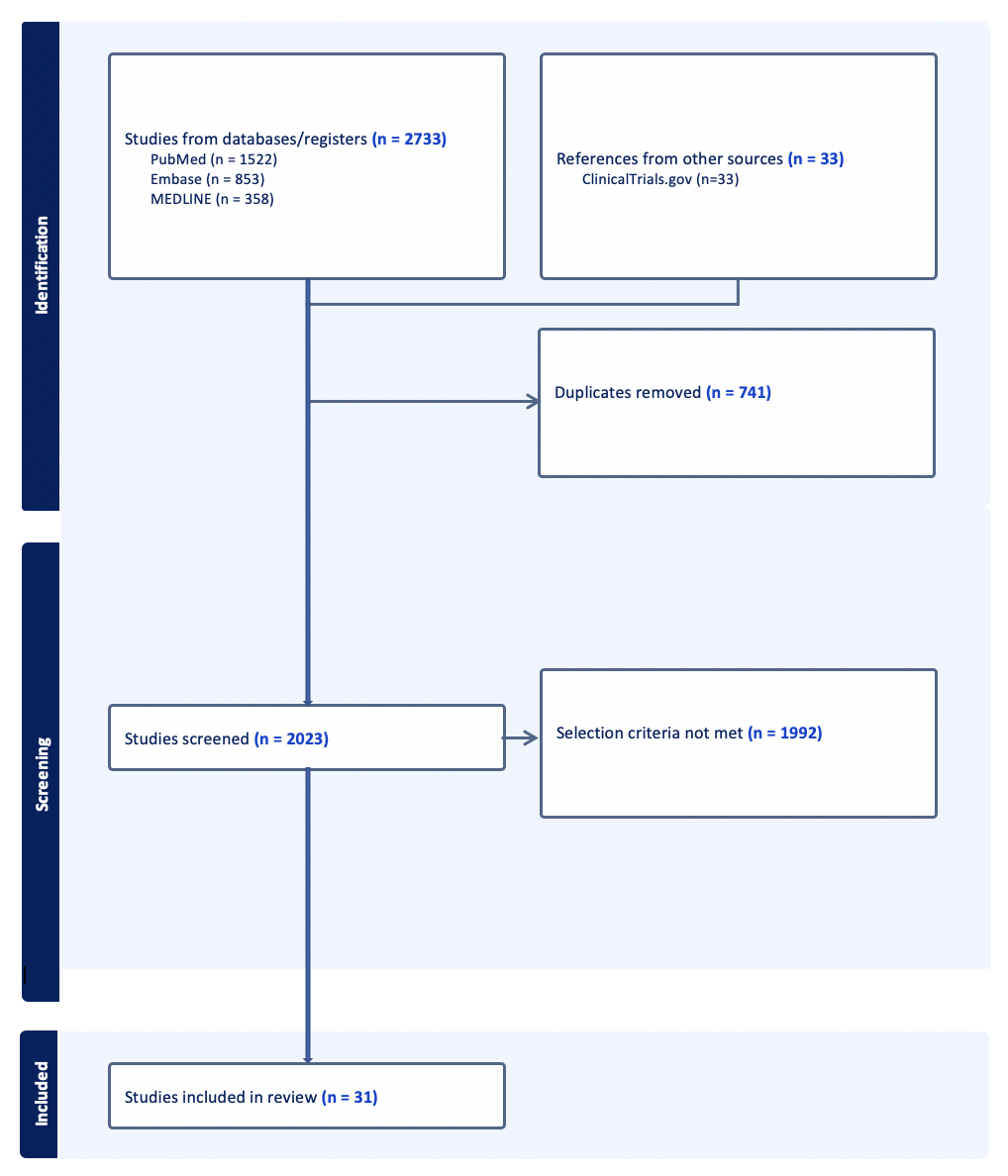


**Supplementary Table 3**: A full list of extracted parameters^1^.

For pre-clinical Phase 0 studies, the following parameters were extracted:
A) Were all predictable risks to patients investigated before human studies began?

B) Were guidelines on best scientific practice and ethics specific to the types of study followed where available?

C) Was a minimum dataset describing technical consistency made public before first-in-human testing?

D) Did the outcome description address relevant parameters:

- Whether the intended goal of procedure is accomplished?
- Level of difficulty of performing procedure or using device as compared to standard of care?
- Safety risks?
- Desirability of intervention?

E) Was the stage endpoint reached? (Any studies that could avoid predictable risks of failure or harm to the first human should have been conducted.)

For clinical Phase 1 studies:
A) Were full details of patient selection, technique and outcomes and patients not selected during the time frame, and why provided?

B) Were standard well-defined measures for reporting outcome and patient characteristics used?

C) Was a structured reporting system used?

D) Was the above information made available to peers regardless of outcome?

E) Was the stage endpoint reached? (Outcomes will determine whether to proceed to stage 2a.)

For clinical Phase 1/2a studies:
A) Was a study protocol made available?

B) Were standard well-defined measures for reporting outcome and patient characteristics used?

C) Were all exclusions reported and explained?

D) Were all cases reported sequentially with annotation and explanation of when and why changes to indication or procedure took place?

E) Was stage endpoint reached? (Display main outcomes graphically to illustrate the above.)

Furthermore, IDEAL-D recommends the use of general ethical best practise for all studies, regardless of stage. We used the following additional parameters to examine ethical conduction of the studies:

1. Transparency: study investigators should be transparent about the methods and results of their research. This includes disclosing any potential conflicts of interest and providing clear and accurate information to study participants.
2. Patient Safety: the safety and well-being of study participants should be the top priority throughout the research process. Study investigators should take all necessary steps to ensure that participants are not put at unnecessary risks.
3. Autonomy of patients: Study investigators should respect the autonomy and dignity of study participants. This includes obtaining informed consent, protecting privacy in terms of data sharing, and ensuring that participants are treated with respect and sensitivity.
4. Animal safety and welfare: Researchers should treat animals with respect and dignity. This includes minimizing any harm or suffering experienced by animals during the study and ensuring that animals are euthanized in a humane manner on study conclusion. Animals used should be provided with appropriate housing, food, water, and medical care. The "3Rs" framework encourages researchers to replace the use of animals with alternative methods where possible, reduce the number of animals used in a study, and refine the experimental design to minimize animal suffering.
5. Ethical board permission: Researchers should report on whether ethical permission was gained for study conduction.

# **Supplementary Table 4**: Scoring on IDEAL-D tool for Phase 0 studies.

| **Author** | A | B | C | D | E |
| --- | --- | --- | --- | --- | --- |
| Mehier-Humbert S et al. (2005)^2^ | No | No | Yes | Yes | No |
| Kinoshita M et al. (2006)^3^ | No | Partially | Yes | Yes | No |
| McDannold N et al. (2006)^4^ | No | No | Yes | Yes | No |
| Treat LH et al. (2007)^5^ | No | Partially | Yes | Yes | No |
| Mei J et al. (2009)^6^ | No | Partially | Yes | Yes | No |
| Liu Y et al. (2010)^7^ | No | Partially | Yes | Yes | No |
| Liu H-L et al. (2010)^8^ | Yes | Partially | Yes | Yes | Partially |
| McDannold N et al. (2012)^9^ | Yes | Partially | Yes | Yes | Partially |
| Ziadloo A et al. (2013)^10^ | Yes | Partially | Yes | Yes | Partially |
| Hsu PH et al. (2013)^11^ | No | Partially | Yes | Yes | No |
| Wei KC et al. (2013)^12^ | No | Partially | Yes | Yes | No |
| Alonso A et al. (2013)^13^ | No | Yes | Yes | Yes | Partially |
| Fan C-H et al. (2015)^14^ | No | No | Yes | Yes | No |
| Chen PY et al. (2015)^15^ | No | Partially | Yes | Yes | Partially |
| McDannold N et al. (2019)^16^ | Yes | Partially | Yes | Yes | Partially |

A) Were all predictable risks to patients investigated before human studies began?

B) Were guidelines on best scientific practice and ethics specific to the types of study followed where available?

C) Was a minimum dataset describing technical consistency made public before first-in-human testing?

D) Did the outcome description address relevant parameters:

- Whether the intended goal of procedure is accomplished?
- Level of difficulty of performing procedure or using device as compared to standard of care?
- Safety risks?
- Desirability of intervention?

E) Was the stage endpoint reached? (Any studies that could avoid predictable risks of failure or harm to the first human should have been conducted.)

# **Supplementary Table 5**: Scoring on SYRCLE tool for Phase 0 studies^17^.

| **Author** | D1 | D2 | D3 | D4 | D5 | D6 | D7 | D8 | D9 | D10 | D11 |
| --- | --- | --- | --- | --- | --- | --- | --- | --- | --- | --- | --- |
| Mehier-Humbert S et al. (2005) | HIGH | HIGH | HIGH | UNCLEAR | HIGH | UNCLEAR | HIGH | UNCLEAR | LOW | HIGH | HIGH |
| Kinoshita M et al. (2006) | UNCLEAR | LOW | UNCLEAR | UNCLEAR | UNCLEAR | UNCLEAR | UNCLEAR | LOW | LOW | UNCLEAR | UNCLEAR |
| McDannold N et al. (2006) | UNCLEAR | LOW | HIGH | UNCLEAR | HIGH | UNCLEAR | HIGH | HIGH | LOW | HIGH | HIGH |
| Treat LH et al. (2007) | UNCLEAR | LOW | UNCLEAR | UNCLEAR | UNCLEAR | UNCLEAR | UNCLEAR | LOW | LOW | UNCLEAR | UNCLEAR |
| Mei J et al. (2009) | UNCLEAR | LOW | UNCLEAR | UNCLEAR | UNCLEAR | UNCLEAR | UNCLEAR | LOW | LOW | UNCLEAR | UNCLEAR |
| Liu Y et al. (2010) | UNCLEAR | LOW | UNCLEAR | UNCLEAR | UNCLEAR | HIGH | UNCLEAR | LOW | LOW | HIGH | HIGH |
| Liu H-L et al. (2010) | UNCLEAR | LOW | UNCLEAR | UNCLEAR | UNCLEAR | UNCLEAR | UNCLEAR | LOW | LOW | LOW | LOW |
| McDannold N et al. (2012) | UNCLEAR | LOW | UNCLEAR | UNCLEAR | UNCLEAR | UNCLEAR | UNCLEAR | LOW | LOW | LOW | LOW |
| Ziadloo A et al. (2013) | LOW | LOW | UNCLEAR | UNCLEAR | UNCLEAR | UNCLEAR | UNCLEAR | LOW | LOW | LOW | LOW |
| Hsu PH et al. (2013) | UNCLEAR | UNCLEAR | UNCLEAR | UNCLEAR | UNCLEAR | UNCLEAR | UNCLEAR | LOW | LOW | UNCLEAR | UNCLEAR |
| Wei KC et al. (2013) | UNCLEAR | UNCLEAR | UNCLEAR | UNCLEAR | UNCLEAR | UNCLEAR | UNCLEAR | LOW | LOW | UNCLEAR | UNCLEAR |
| Alonso A et al. (2013) | UNCLEAR | LOW | UNCLEAR | UNCLEAR | LOW | UNCLEAR | LOW | LOW | LOW | LOW | LOW |
| Fan C-H et al. (2015) | LOW | LOW | UNCLEAR | UNCLEAR | UNCLEAR | HIGH | HIGH | LOW | LOW | UNCLEAR | HIGH |
| Chen PY et al. (2015) | UNCLEAR | LOW | UNCLEAR | UNCLEAR | UNCLEAR | UNCLEAR | UNCLEAR | LOW | LOW | UNCLEAR | UNCLEAR |
| McDannold N et al. (2019) | LOW | LOW | UNCLEAR | UNCLEAR | UNCLEAR | UNCLEAR | LOW | LOW | LOW | HIGH | UNCLEAR |

D1: RANDOM SEQUENCE GENERATION

Was the allocation sequence adequately generated and applied?

D2: BASELINE CHARACTERISTICS

Were the groups similar at baseline or were they adjusted for confounders in the analysis?

D3: ALLOCATION CONCEALMENT

Was the allocation adequately concealed?

D4: RANDOM HOUSING

Were the animals randomly housed during the experiment?

D5: BLINDING

Were the caregivers and/or investigators blinded from knowledge which intervention each animal received during the experiment?

D6: RANDOM OUTCOME ASSESSMENT

Were animals selected at random for outcome assessment?

D7: BLINDING

Was the outcome assessor blinded?

D8: INCOMPLETE OUTCOME DATA

Were incomplete outcome data adequately addressed?

D9: SELECTIVE OUTCOME REPORTING

Are reports of the study free of selective outcome reporting?

D10: OTHER SOURCES OF BIAS

Sample size calculations done?

D11: OVERALL SCORE

Of the 15 studies evaluated, only three studies were found to have a low risk of bias for sequence generation as they addressed randomization of experimental samples against controls. On the other hand, 12 studies were categorized as having "some concerns" or unclear risk of bias due to not addressing randomization of samples at all. One study^2^ scored highly on sequence generation as it did not employ any controls. Most of the studies (n=12/15) scored as low risk of bias for baseline characteristics. However, two studies scored as "some concerns" due to insufficient in-depth information on animals, such as not mentioning weight and race. Mehier-Humbert S et al.^2^ scored highly on this parameter because they did not provide basic information on the mice samples used. In terms of allocation concealment, most studies (n=13/15) scored as "some concerns" due to lack of mention of it. Mehier-Humbert S et al.^2^ scored highly due to the absence of controls, which made it impossible to conceal allocation. McDannold N et al.^4^ also scored highly due to one person performing histology, radiological evaluation, and statistical analysis of all results. All studies (n=15/15) scored "some concerns" for random housing as they did not address it at all. Regarding blinding of caregivers, most studies (n=12/15) did not address it. Mehier-Humbert S et al. ^2^ and McDannold N et al.^4^ scored highly for this parameter due to the same reasons. Only Alonso A et al.^13^ scored "low risk of bias" due to their double-blinding methodology. Thirteen studies received an unclear risk of bias score for random outcome assessment as the methods used were not mentioned. Fan C-H et al.^14^ employed a histologist who was informed of the sonicated side, hence both studies were scored as having high risk of bias. Most studies (n=10/15) did not address outcome assessor blinding, and hence they scored unclear risk of bias. Mehier-Humbert S et al.^2^ and McDannold N et al.^4^ scored highly for this parameter for the same reasons. Only Alonso A et al.^13^ and McDannold N et al.^16^ explicitly mentioned outcome blinding. Most studies were deemed to have low risk of bias for incomplete outcome data (n=13/15), while McDannold N et al.^4^ scored highly due to not describing data recorded for outliers. All studies were found to have low risk of selective outcome reporting (n=15/15). In terms of other sources of bias, most were sequelae or related sources of bias to the lack of controls, blinding, and sample size calculations. Overall, four studies were scored as "high risk of bias", seven studies as "unclear risk of bias", and four studies as "low risk of bias".

# **Supplementary Table 6**: Scoring on IDEAL-D tool for Phase 1 studies.

| **Author** | **A** | **B** | **C** | **D** | **E** |
| --- | --- | --- | --- | --- | --- |
| Mainprize, T et al. (2019)^18^ | Partially | Yes | Yes | Yes | Partially |
| Idbaih A et al. (2019)^19^ | Partially | Yes | Yes | Yes | Partially |

# **Supplementary Table 7**: Scoring on ROBINS-1 tool for Phase 1 studies^20^.

| **Author** | D1 | D2 | D3 | D4 | D5 | D6 | D7 | D8 |
| --- | --- | --- | --- | --- | --- | --- | --- | --- |
| Mainprize, T et al. (2019) | HIGH | LOW | MODERATE | LOW | HIGH | HIGH | LOW | HIGH |
| Idbaih A et al. (2019) | HIGH | LOW | LOW | LOW | LOW | HIGH | LOW | MODERATE |

D1: Bias due to confounding

D2: Bias in selection of participants into the study

D3: Bias in classification of interventions

D4: Bias due to deviations from intended interventions

D5: Bias due to missing data

D6: Bias in measurement of outcomes

D7: Bias in selection of the reported result

D8: Overall bias

# **Supplementary Table 8**: Scoring on IDEAL-D tool for Phase 1 studies.

| **Author** | **A** | **B** | **C** | **D** | **E** |
| --- | --- | --- | --- | --- | --- |
| Park SH et al. (2020)^21^ | Yes | No | Yes | Yes | No |
| Carpentier A et al. (2016)^22^ | Yes | No | Yes | Yes | No |

# **Supplementary Table 9**: Scoring on ROBINS-1 tool for Phase 1 studies.

| **Author** | **D1** | **D2** | **D3** | **D4** | **D5** | **D6** | **D7** | **D8** |
| --- | --- | --- | --- | --- | --- | --- | --- | --- |
| Park SH et al. (2020) | LOW | LOW | LOW | LOW | LOW | HIGH | MODERATE | MODERATE |
| Carpentier A et al. (2016) | LOW | LOW | LOW | LOW | LOW | HIGH | MODERATE | MODERATE |

D1: Bias due to confounding

D2: Bias in selection of participants into the study

D3: Bias in classification of interventions

D4: Bias due to deviations from intended interventions

D5: Bias due to missing data

D6: Bias in measurement of outcomes

D7: Bias in selection of the reported result

D8: Overall bias

# **References**

1. Hirst A, Philippou Y, Blazeby J, et al. No Surgical Innovation Without Evaluation: Evolution and Further Development of the IDEAL Framework and Recommendations. *Ann Surg*. 2019;269(2):211-220. doi:10.1097/SLA.0000000000002794

2. Mehier-Humbert S, Bettinger T, Yan F, Guy RH. Plasma membrane poration induced by ultrasound exposure: Implication for drug delivery. *Journal of Controlled Release*. 2005;104(1):213-222. doi:10.1016/J.JCONREL.2005.01.007

3. Kinoshita M, McDannold N, Jolesz FA, Hynynen K. Noninvasive localized delivery of Herceptin to the mouse brain by MRI-guided focused ultrasound-induced blood-brain barrier disruption. *Proc Natl Acad Sci U S A*. 2006;103(31):11719-11723. doi:10.1073/PNAS.0604318103/SUPPL_FILE/04318FIG4.PDF

4. McDannold N, Vykhodtseva N, Hynynen K. Targeted disruption of the blood–brain barrier with focused ultrasound: association with cavitation activity. *Phys Med Biol*. 2006;51(4):793. doi:10.1088/0031-9155/51/4/003

5. Treat LH, McDannold N, Vykhodtseva N, Zhang Y, Tam K, Hynynen K. Targeted delivery of doxorubicin to the rat brain at therapeutic levels using MRI-guided focused ultrasound. *Int J Cancer*. 2007;121(4):901-907. doi:10.1002/IJC.22732

6. Mei J, Cheng Y, Song Y, et al. Experimental Study on Targeted Methotrexate Delivery to the Rabbit Brain via Magnetic Resonance Imaging–Guided Focused Ultrasound. *Journal of Ultrasound in Medicine*. 2009;28(7):871-880. doi:10.7863/JUM.2009.28.7.871

7. Liu HL, Hua MY, Yang HW, et al. Magnetic resonance monitoring of focused ultrasound/magnetic nanoparticle targeting delivery of therapeutic agents to the brain. *Proc Natl Acad Sci U S A*. 2010;107(34):15205-15210. doi:10.1073/PNAS.1003388107/SUPPL_FILE/PNAS.201003388SI.PDF

8. Liu HL, Hua MY, Chen PY, et al. Blood-brain barrier disruption with focused ultrasound enhances delivery of chemotherapeutic drugs for glioblastoma treatment. *Radiology*. 2010;255(2):415-425. doi:10.1148/RADIOL.10090699/ASSET/IMAGES/LARGE/10090699FIG08F.JPEG

9. McDannold N, Arvanitis CD, Vykhodtseva N, Livingstone MS. Temporary disruption of the blood-brain barrier by use of ultrasound and microbubbles: Safety and efficacy evaluation in rhesus macaques. *Cancer Res*. 2012;72(14):3652-3663. doi:10.1158/0008-5472.CAN-12-0128/650292/AM/TEMPORARY-DISRUPTION-OF-THE-BLOOD-BRAIN-BARRIER-BY

10. Ziadloo A, Burks SR, Gold EM, et al. Enhanced Homing Permeability and Retention of Bone Marrow Stromal Cells by Noninvasive Pulsed Focused Ultrasound. *Stem Cells*. 2012;30(6):1216-1227. doi:10.1002/STEM.1099

11. Hsu PH, Wei KC, Huang CY, et al. Noninvasive and Targeted Gene Delivery into the Brain Using Microbubble-Facilitated Focused Ultrasound. *PLoS One*. 2013;8(2):e57682. doi:10.1371/JOURNAL.PONE.0057682

12. Wei KC, Chu PC, Wang HYJ, et al. Focused Ultrasound-Induced Blood–Brain Barrier Opening to Enhance Temozolomide Delivery for Glioblastoma Treatment: A Preclinical Study. *PLoS One*. 2013;8(3):e58995. doi:10.1371/JOURNAL.PONE.0058995

13. Alonso A, Reinz E, Leuchs B, et al. Focal Delivery of AAV2/1-transgenes Into the Rat Brain by Localized Ultrasound-induced BBB Opening. *Mol Ther Nucleic Acids*. 2013;2:e73. doi:10.1038/MTNA.2012.64

14. Fan CH, Ting CY, Chang YC, Wei KC, Liu HL, Yeh CK. Drug-loaded bubbles with matched focused ultrasound excitation for concurrent blood–brain barrier opening and brain-tumor drug delivery. *Acta Biomater*. 2015;15:89-101. doi:10.1016/J.ACTBIO.2014.12.026

15. Chen PY, Hsieh HY, Huang CY, Lin CY, Wei KC, Liu HL. Focused ultrasound-induced blood-brain barrier opening to enhance interleukin-12 delivery for brain tumor immunotherapy: A preclinical feasibility study. *J Transl Med*. 2015;13(1):1-12. doi:10.1186/S12967-015-0451-Y/TABLES/1

16. McDannold N, Zhang Y, Supko JG, et al. Acoustic feedback enables safe and reliable carboplatin delivery across the blood-brain barrier with a clinical focused ultrasound system and improves survival in a rat glioma model. *Theranostics*. 2019;9(21):6284. doi:10.7150/THNO.35892

17. Hooijmans CR, Rovers MM, De Vries RBM, Leenaars M, Ritskes-Hoitinga M, Langendam MW. SYRCLE’s risk of bias tool for animal studies. *BMC Med Res Methodol*. 2014;14(1):43. doi:10.1186/1471-2288-14-43

18. Mainprize T, Lipsman N, Huang Y, et al. Blood-Brain Barrier Opening in Primary Brain Tumors with Non-invasive MR-Guided Focused Ultrasound: A Clinical Safety and Feasibility Study. *Scientific Reports 2019 9:1*. 2019;9(1):1-7. doi:10.1038/s41598-018-36340-0

19. Idbaih A, Canney M, Belin L, et al. Safety and feasibility of repeated and transient blood-brain barrier disruption by pulsed ultrasound in patients with recurrent glioblastoma. *Clinical Cancer Research*. 2019;25(13):3793-3801. doi:10.1158/1078-0432.CCR-18-3643/74166/AM/SAFETY-AND-FEASIBILITY-OF-REPEATED-AND-TRANSIENT

20. Sterne JA, Hernán MA, Reeves BC, et al. ROBINS-I: A tool for assessing risk of bias in non-randomised studies of interventions. *BMJ (Online)*. 2016;355. doi:10.1136/bmj.i4919

21. Park SH, Kim MJ, Jung HH, et al. Safety and feasibility of multiple blood-brain barrier disruptions for the treatment of glioblastoma in patients undergoing standard adjuvant chemotherapy. *J Neurosurg*. 2020;134(2):475-483. doi:10.3171/2019.10.JNS192206

22. Carpentier A, Canney M, Vignot A, et al. Clinical trial of blood-brain barrier disruption by pulsed ultrasound. *Sci Transl Med*. 2016;8(343). doi:10.1126/SCITRANSLMED.AAF6086/SUPPL_FILE/8-343RE2_SM.PDF
